# Elevated Vibration Perception Thresholds in CIDP Patients Indicate More Severe Neuropathy and Lower Treatment Response Rates

**DOI:** 10.1371/journal.pone.0139689

**Published:** 2015-11-06

**Authors:** Alon Abraham, Hana Albulaihe, Majed Alabdali, Mohammad Qrimli, Ari Breiner, Carolina Barnett, Hans D. Katzberg, Leif E. Lovblom, Bruce A. Perkins, Vera Bril

**Affiliations:** 1 Ellen and Martin Prosserman Centre for Neuromuscular Diseases, Division of Neurology, Department of Medicine, University Health Network, University of Toronto, Toronto, Canada; 2 Department of Neurology, King Khalid University Hospital, King Saud University, Riyadh, Saudi Arabia; 3 Department of Neurology, King Fahad Hospital of the University, University of Dammam, Dammam, Saudi Arabia; 4 Division of Endocrinology and Metabolism, Department of Medicine, Mount Sinai, Hospital and Lunenfeld Tanenbaum Research Institute, University of Toronto, Toronto, Canada; University of Würzburg, GERMANY

## Abstract

**Introduction:**

Vibration perception threshold (VPT) examination using a neurothesiometer provides objective, sensitive and specific information, and has been utilized mainly in patients with diabetic polyneropathy.

**Objectives:**

Explore the utility of VPT examination in CIDP patients.

**Methods:**

CIDP subjects attending the Neuromuscular clinic between 01/2013 and 12/2014 were evaluated. Demographic data, clinical history, physical examination, VPT values, and electrophysiologic data from their charts were extracted.

**Results:**

70 charts were reviewed. 55 CIDP patients had elevated VPT, associated with higher frequency of abnormal sensory testing for various modalities (92.7% vs. 46.7%, p<0.0001), lower sensory and motor amplitudes and reduced conduction velocities on nerve conduction studies, and lower treatment response rates (54% vs. 93%, p = 0.01).

**Conclusion:**

VPT examination is a simple tool, which is a reliable and sensitive measure not only for diabetic neuropathy, but also for CIDP. Moreover, in CIDP, elevated VPT values are also associated with lower treatment response rates.

## Introduction

Sensory system evaluation is an important part of the neurological examination. However, it might be occasionally challenging, as it depends on patient cooperation. Primary sensory modalities commonly examined include light touch, pain, temperature, proprioception and vibration sensation. There are 2 different approaches for sensory testing. The first is comparative, identifying relative differences between sites (e.g. distal and proximal), or qualitative (e.g. presence or absence of vibration sensation), and the second is absolute, attempting to grade thresholds objectively[1]. Absolute sensory testing is useful in medical practice, and can provide sensitive and reliable neuropathic endpoints for epidemiological studies and therapeutic trials[2]. Vibration perception thresholds (VPT) examination, is an example of absolute sensory testing, using different devices, such as a neurothesiometer, or case IV device, and provides more objective information, and is more sensitive and specific for detecting changes in sensory nerve function, than qualitative clinical vibration sense testing (VQT). VQT is commonly performed as part of the routine neurological examination, and usually provides limited information for the presence or absence of vibration sensation only[3], although a semi qualitative vibration sense testing also exists, using a graduated tuning fork. Similar to VQT, VPT testing is simple, quick to perform using some tests paradigms, as methods of limits[4], painless and is generally well tolerated[5].

Chronic inflammatory demyelinating polyneuropathy (CIDP) is an immune-mediated neuropathy characterized by motor greater than sensory, proximal and distal peripheral neuropathy, with a slowly progressive or relapsing course. Subjective sensory disturbance is present in 68–80% of CIDP patients, with abnormal sensory examination in most, particularly for large-fiber modalities, such as vibration and proprioception[6]. Although VPT has proven to be a reliable measure of confirmed clinical neuropathy and abnormal nerve conductions studies (NCS) in patients with diabetic polyneuropathy[5], there is no literature regarding its utility in CIDP patients, namely correlation to clinical and electrophysiological testing, and the association with treatment response rates.

## Materials and Methods

### Subjects

CIDP subjects attending the Neuromuscular clinic for management of their immune-mediated polyneuropathy between 01/2013 and 12/2014 were evaluated for this study. In this retrospective review we extracted demographic data, clinical history, physical examination, and electrophysiologic data from their charts. The Research Ethics Board of the University Health Network approved the current study protocol, based on chart review and collection of de-identified data.

CIDP was diagnosed based on the clinical presentation, as judged by a neuromuscular expert (VB), and the presence of demyelination on NCS, as per the Koski criteria[7]. Fulfilling EFNS/PNS CIDP criteria was defined for the purpose of this study as fulfilling electrophysiological criteria only for definite CIDP, excluding probable and possible diagnoses. Subjects were evaluated by clinical history, neurological examination (including VQT), vibration perception thresholds (VPT), and NCS.

Treatment responsiveness was assessed by combining data from the clinical history, neurological examination, and the electrodiagnostic tests results, which was performed as part of the routine follow up evaluation. Treatment responders were defined as those who stabilized after declining progressively, or improved after treatment, whereas treatment non-responders were defined as those who either worsened or did not change after treatment.

Abnormal VQT was defined as absence vibration perception at the toes, using a standard 128 Hz tuning fork, as part of the routine neurological examination.

VPT testing was performed with a Neurothesiometer, using the method of limits[8]. The stimulus was applied to the distal pulp of the first finger and toe on each side, and the patient was requested to indicate when vibration sensation was first perceived. Stimulus strength was gradually increased from null intensity to a value in voltage at which the subject first detected vibration. Testing was carried out with the subject’s eyes closed. Three separate tests were conducted, and a mean of the three values was calculated in volts. A ‘null stimulus’ trial was added randomly to ensure the subject’s adherence and understanding. Testing generally required less than 3 min. Normal values were considered as 5 volts or less in the fingers, and 15 volts or less in the toes[9].

NCS were performed using the Sierra Wave instrument (Cadwell Laboratories Inc., Kennewick, WA, USA). Age- and height-adjusted NCS reference values were used, according to the standards of the Toronto General Hospital (University Health Network) electrophysiology laboratory. Limb temperature was measured prior to nerve conduction studies, and if required, warming was performed to ensure a surface temperature of ≥32.0°C in the hands and ≥31.0°C in the feet.

Median, ulnar, peroneal, tibial and sural NCS were performed using surface stimulating and recording techniques according to the standards of the Canadian Society of Clinical Neurophysiology and the American Association of Neuromuscular and Electrodiagnostic Medicine[10]. The electromyography Instrument calculated latencies, amplitudes and conduction velocities automatically. Median, peroneal and tibial nerve compound motor action potential (CMAP) amplitudes were measured from first negative peak to the next positive peak. Median, and sural sensory nerve action potential (SNAP) amplitudes were also measured from the first positive peak if present, to the next positive peak

### Statistical analysis

Statistical analysis was performed using SAS version 9.2 for Windows (SAS Institute, Cary, North Carolina). Clinical and electrophysiological characteristics were expressed as mean ± standard deviation (SD) for continuous variables, or as frequency and percent for ordinal variables. Comparisons between groups were made using the student’s t-test, the Wilcoxon rank-sum test, or the χ2-test, depending on the type and distribution of the variable. Receiver operating characteristic (ROC) curves for the identification of abnormal NCS parameters by VPT were generated as follows: At each upper limb site (the median sensory nerve and median motor nerve), VPT measured at the finger was the continuous “test variable”, and a separate ROC curve was generated for two dichotomous outcomes, one being abnormal amplitude, and the other being abnormal conduction velocity. Likewise, at each lower limb site (the sural, tibial, and peroneal nerves), VPT measured at the toe was the continuous test variable, and abnormal conduction velocity and abnormal amplitude were the dichotomous outcome variables. Areas under the ROC curve (AUC) were calculated, and optimal operating thresholds for each test and outcome combination were chosen so as to maximize specificity while retaining high sensitivity. Additionally, VQT was treated as a dichotomous test for identifying the same abnormal NCS parameters. P-values less than 0.05 were considered statistically significant.

## Results

70 CIDP patients’ charts were reviewed. Their demographic data is shown in Table 1. 55 CIDP patients had elevated VPT, while 15 patients had normal values. Patients with elevated VPT were older (62±11 vs. 52±11, p = 0.002), with male predominance (84% vs. 47%, p = 0.003). Abnormal sensory testing for various modalities on neurological examination was more frequent in CIDP patients with elevated VPT at the toes (92.7% vs. 46.7%, p<0.0001), including VQT (92.7% vs. 46.7%), proprioception (50.9% vs. 13.3%, p = 0.009), and pain perception (83.6% vs. 46.7%, p = 0.003) testing, with a trend toward distal lower limb weakness (40% vs. 13%, p = 0.05). CIDP patients with elevated finger VPT had lower median nerve sensory and motor amplitudes (15.5±21.4 vs. 31.5±25.5 μV, p = 0.0004, and 8.7±5.3 vs. 12.0±5.4 mV, p = 0.03), as well as reduced sensory conduction velocities (40.0±12.1 vs. 46.4±9.8 m/s, p = 0.02). CIDP patients with elevated toe VPT had also lower sural, peroneal and tibial nerves amplitudes (2.6±3.1 vs. 7.6±5.7 μV, p = 0.0003, 2.4±2.1 vs. 4.6±3.5 mV, p = 0.01, and 4.3±5.8 vs. 12.4±6.7 mV, p = <0.0001 correspondingly), and reduced conduction velocities (34.0±4.6 vs. 42.3±5.6 m/s, p<0.0001, 32.7±6.5 vs. 39.5±5.8 m/s, p = 0.0006, and 30.4±7.6 vs. 37.7±7.4 m/s, p = 0.002 correspondingly). Although a higher number of CIDP patients with elevated VPT fulfilled EFNS/PNS electrophysiological criteria for CIDP (51% vs. 13%, p = 0.01), treatment response rates were lower significantly in this group (54% vs. 93%, p = 0.01). Similar responder rate are shown after excluding CIDP patients with diabetes (54% vs. 92%, p = 0.03) (Table 2). Similar results are obtained by VQT (Table 3).

**Table 1:** Clinical characteristics of CIDP patients with normal and abnormal VPT.

	Total Cohort	Normal VPT	Elevated VPT	p-value*
n	70	15	55	
Age (years)	60±12	52±11	62±11	0.002
Male sex	53 (76%)	7 (47%)	46 (84%)	0.003
Diabetes	22 (32%)	2 (13%)	20 (37%)	0.08
Hypertension	29 (43%)	5 (33%)	24 (46%)	0.38
Hyperlipidemia	27 (57%)	4 (50%)	23 (59%)	0.64
Thyroid disease	6 (9%)	2 (13%)	4 (8%)	0.52
Smoking	23 (34%)	7 (47%)	16 (31%)	0.25
Alcohol	2 (3%)	0	2 (4%)	0.44
CIDP duration (years)	8±7	11±11	7±5	0.17
Weakness distribution				
Symmetrical	55 (79%)	13 (87%)	42 (76%)	0.39
Upper limbs	49 (70%)	13 (86%)	36 (66%)	0.11
Lower limbs	69 (99%)	15 (100%)	54 (98%)	0.60
Proximal leg weakness	24 (34%)	6 (40%)	18 (33%)	0.60
Distal leg weakness	24 (34%)	2 (13%)	22 (40%)	0.05
Sensory deficit				
Light touch	29 (41%)	6 (40%)	23 (41.8%)	0.90
Proprioception	30 (43%)	2 (13.3%)	28 (50.9%)	0.009
Vibration	58 (83%)	7 (46.7%)	51 (92.7%)	<0.0001
Temperature	50 (71%)	8 (53.3%)	42 (76.4%)	0.08
Pain	53 (76%)	7 (46.7%)	46 (83.6%)	0.003
VPT				
Finger	6.75±5.62	3.71±0.89	7.58±6.08	<0.0001
Toe	25.16±12.93	9.44±3.82	29.45±11.06	<0.0001

For categorical variables, the results are given as frequency and percent. For continuous variables, the results include mean and standard deviation.

VPT—vibration perception thresholds

CIDP—Chronic inflammatory demyelinating polyneuropathy

**Table 2:** Electrophysiological characteristics of CIDP patients with normal and abnormal VPT.

	Total Cohort	Normal VPT	Elevated VPT	p-value*
Nerve conduction parameters				
Median sensory				
Amplitude (μV)	24.07±24.85	31.5±25.5	15.5±21.4	0.0004
Velocity (m/s)	43.45±11.30	46.4±9.8	40.0±12.1	0.02
Median motor				
Amplitude (mV)	10.46±5.59	12.0±5.4	8.7±5.3	0.03
Velocity (m/s)	44.32±9.58	46.5±7.3	41.8±11.3	0.08
Sural				
Amplitude (μV)	3.89±4.49	7.6±5.7	2.6±3.1	0.0003
Velocity (m/s)	36.19±6.09	42.3±5.6	34.0±4.6	<0.0001
Peroneal				
Amplitude (mV)	3.11±2.78	4.6±3.5	2.4±2.1	0.01
Velocity (m/s)	34.52±6.97	39.5±5.8	32.7±6.5	0.0006
Tibial				
Amplitude (mV)	6.37±6.97	12.4±6.7	4.3±5.8	<0.0001
Velocity (m/s)	32.27±8.16	37.7±7.4	30.4±7.6	0.002
EFNS criteria	30 (43%)	2 (13%)	28 (51%)	0.01
Responders, n (%)	35 (64%)	13 (93%)	22 (54%)	0.01
Responders*, n (%)	25 (52%)	11 (92%)	14 (54%)	0.03

For categorical variables, the results are given as frequency and percent. For continuous variables, the results include mean and standard deviation

EFNS—European Federation of Neurological Societies

VPT—vibration perception thresholds

CIDP—Chronic inflammatory demyelinating polyneuropathy

Responders*—excluding patients with diabetes.

**Table 3:** Electrophysiological characteristics and treatment response rates in the presence of normal and abnormal vibration manual testing in CIDP patients.

	Total Cohort	Normal VQT	Elevated VQT	p-value*
Sural				
Amplitude (μV)	3.89±4.49	8.2±6.1	3.0±3.5	0.002
Velocity (m/s)	36.19±6.09	42.7±5.8	34.8±5.2	0.0002
Peroneal				
Amplitude (mV)	3.11±2.78	4.5±2.8	2.7±2.7	0.02
Velocity (m/s)	34.52±6.97	38.9±5.2	33.6±7.0	0.02
Tibial				
Amplitude (mV)	6.37±6.97	11.6±7.2	5.1±6.4	0.004
Velocity (m/s)	32.27±8.16	36.5±8.3	31.3±7.9	0.06
EFNS criteria	30 (43%)	3 (25%)	27 (47%)	0.17
Responders	35 (64%)	9 (100%)	26 (57%)	0.01

VQT—qualitative clinical vibration sense testing

CIDP—Chronic inflammatory demyelinating polyneuropathy

EFNS—European Federation of Neurological Societies

Similar associations were observed in CIDP patients with abnormal VQT, showing high sensitivity for detecting lower limb sensory and motor nerve conduction abnormalities, and lower treatment response rates (57% vs. 100%, p = 0.01). However, VQT was less specific compared to VPT testing (Table 4).

**Table 4:** Sensitivities and Specificities of Vibration perception thresholds and vibration manual testing for abnormal nerve conduction studies.

	VPT		VQT	
	Sensitivity	Specificity	Sensitivity	Specificity
Sural				
Amplitude (μV)	82%	57%	89%	43%
Velocity (m/s)	90%	68%	94%	47%
Peroneal				
Amplitude (mV)	75%	50%	84%	36%
Velocity (m/s)	81%	64%	86%	36%
Tibial				
Amplitude (mV)	87%	59%	91%	47%
Velocity (m/s)	83%	64%	87%	45%

VPT—vibration perception thresholds

VQT—qualitative clinical vibration sense testing

**Fig 1:**
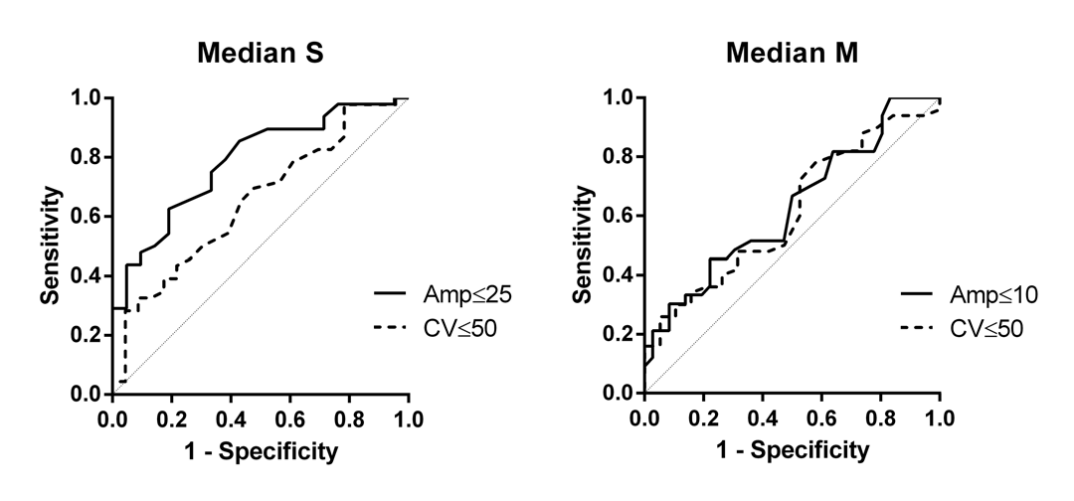
VPT Finger. AUC for Median S Amp = 0.79 and Median S CV = 0.66. AUC for Median M Amp = 0.63 and Median M CV = 0.61.

**Fig 2:**
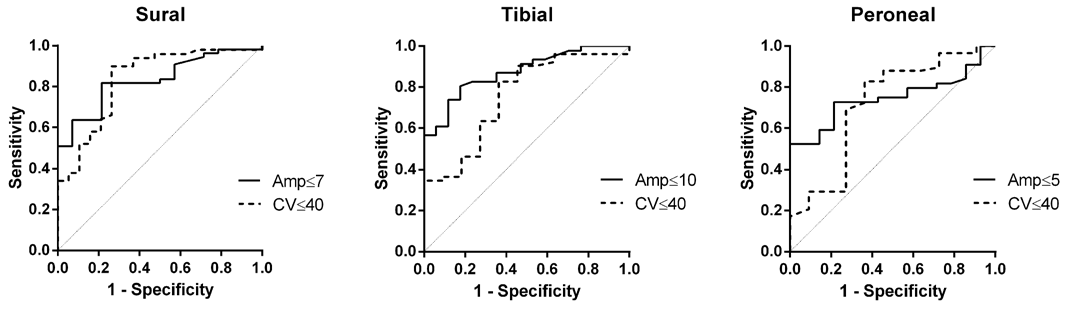
VPT Toe. AUC for Sural Amp = 0.83 and Sural CV = 0.84. AUC for Peroneal Amp = 0.75 and Peroneal CV = 0.72. AUC for Tibial Amp = 0.87 and Tibial CV = 0.75.

VPT receiver operating characteristic ROC area under curves were generally high, especially for sural nerve amplitude (0.83), conduction velocity (0.84), and tibial nerve motor amplitude (0.87) (Figs 1 and 2).

## Discussion

The current study shows that elevated VPT in CIDP patients is associated with a more severe neuropathy, manifested by worse clinical and electrophysiological examinations (Tables 1 and 2). Although similar results are obtained by VQT (Table 3), showing high sensitivity for detecting abnormal NCS (around 85%), VQT is less specific compared to VPT (Table 4). High sensitivity of VPT testing is also expressed by high ROC AUC, especially for sural nerve amplitude (0.83), conduction velocity (0.84), and tibial nerve motor amplitude (0.87) (Figs 1 and 2).

As CIDP involves multiple sensory and motor nerves, it is not surprising that abnormal sensory testing in one sensory modality, such as vibration, is associated with sensory abnormalities in other modalities, and even with a trend for distal leg weakness. Similarly, there is electrophysiological evidence not only for worse sensory NCS, but also for worse motor NCS, manifested by reduced amplitudes and lower conduction velocities. These findings imply that VPT testing is a reliable and a sensitive measure for CIDP.

An additional important finding in this study is the association of abnormal vibration testing and lower treatment response rates in CIDP patients. A similar association is shown after excluding CIDP patients with diabetes, although the have higher percentage of abnormal vibration sensation. Although 60 to 80 percent of CIDP patients are expected to respond to treatment, it is difficult to predict treatment responsiveness. Prediction of outcome has been related to the pattern of weakness[11], the presence of monoclonal gammopathy[12], distribution patterns of conduction abnormalities[13,14], the selection of electrodiagnostic criteria[15], and disease duration[16].

Lower treatment response rates in CIDP patients with abnormal vibration perception might be expected at least theoretically, due to a more severe neuropathy, as reflected by worse clinical and electrophysiological findings, which might be associated with more widespread irreversible nerve fiber loss. However, the largest study of IVIG in CIDP patients to date has shown that advanced loss of axons does not lead to failure to respond to treatment[17,18]. Therefore, lower treatment response rates in this cohort of CIDP patients with abnormal vibration testing is surprising, suggesting vibration testing as a unique tool that might assist predicting treatment response rates in CIDP. Nonetheless, it should be emphasized that even in the presence of abnormal VPT, more than half the patients do respond to treatment. As a rule, we used IVIG as a first line treatment for CIDP patients (using a loading dose of 2 g/kg, followed by monthly infusions of 1 g/kg), utilizing additional immunomodulatory treatments in case of treatment failure.

The finding of an older age in CIDP patients with elevated VPT is not surprising, as age affects peripheral nerve function. In addition, higher VPT values were also found among older patients with diabetic polyneuropathy[5]. In contrast, we do not have a satisfying explanation for male predominance in CIDP patients with abnormal VPT in our study. Although age and sex does not affect treatment response rates, fulfilling EFNS/PNS electrophysiological criteria is known to be associated with higher treatment response rates[12,15]. Although a higher number of CIDP patients with abnormal VPT in the current study fulfilled EFNS/PNS electrophysiological criteria for CIDP (51% vs. 13%, p = 0.01), surprisingly they had lower treatment response rates (54% vs. 93%, p = 0.01), implying that predicting treatment responsiveness using EFNS/PNS criteria might be limited. As abnormal VPT were associated with worse NCS, higher frequency for fulfilling EFNS/PNS criteria within these patients is not surprising.

The current study has several limitations. First, although only statistically significant results were considered, the numbers are low, mainly in CIDP patients with normal vibration testing. In addition, misclassification and selection bias are potential errors, as there are no biomarkers to make a definitive diagnosis of CIDP. There are additional neuropathies causing proximal weakness, such as diabetic radiculoplexopathies, and slowing of conduction velocity might be due to loss of ion channels in the inter-nodal region or other factors without true demyelination. Current electrodiagnostic criteria for CIDP also have limited sensitivity, as they are research oriented, favouring specificity over sensitivity [19]. And finally, the lack of reliable biomarkers is not limited only for the diagnosis of CIDP, but also for assessing treatment responsiveness. Although this can be usually done reliably combining information from the clinical history, disability and functional questioners, and from clinical and electrophysiological examinations, misclassification is still a potential concern.

In conclusion, VPT examination is a simple tool, which is a reliable and sensitive measure not only for diabetic neuropathy, but also for evaluating CIDP severity. Moreover, in CIDP elevated VPT are also associated with lower treatment response rates, although still more than half of patients will respond to treatment. Prospective trials are required in order to confirm these findings, and further explore the utility of VPT testing in other neuropathies.

## Supporting Information

S1 Database (XLS)
